# Transverse Sinus Stenting Reverses Medically Refractory Idiopathic Intracranial Hypertension

**DOI:** 10.3389/fopht.2022.885583

**Published:** 2022-06-21

**Authors:** Kate Reid, H. Stephen Winters, Timothy Ang, Geoffrey D. Parker, G. Michael Halmagyi

**Affiliations:** ^1^ Neuro-Ophthalmology Service, Ophthalmology Department, Canberra Hospital, Canberra, ACT, Australia; ^2^ Medical School, Australian National University, Canberra, ACT, Australia; ^3^ Medical Imaging Department, Royal Prince Alfred Hospital, Sydney, NSW, Australia; ^4^ Neurology Department, Royal Prince Alfred Hospital, Sydney, NSW, Australia; ^5^ Central Clinical School, University of Sydney, Sydney, NSW, Australia

**Keywords:** medically refractory, IIH, transverse sinus stenosis, stenting for IIH, cerebral venous hypertension, visual threat in IIH

## Abstract

**Aim:**

To characterise the ophthalmic indications for, and ophthalmic efficacy of, transverse sinus stenting in adults with medically refractory idiopathic intracranial hypertension.

**Methods:**

A retrospective cohort study was undertaken on a single-author database of 226 successive patients with confirmed idiopathic intracranial hypertension (IIH). A total of 32 patients were identified who received a transverse sinus stent for medically refractory disease. This which was defined as visual threat and/or intolerance of maximal medical therapy. Patients with medically refractory disease proceeded to stenting, if found to have a significant transverse sinus stenosis gradient at catheter venography. Visual threat was quantified *via* the degree of papilledema on optical coherence tomography of the retinal nerve fibre layer, and *via* the visual field mean deviation. CSF opening pressure at lumbar puncture and cerebral venous sinus pressure measurements from catheter venography were correlated with the ophthalmic data, noting also intolerance of maximal medical therapy. Complications of stenting were fully assessed.

**Results:**

Medically refractory IIH was found in 18% of the total cohort of IIH patients. 90% of those with medically refractory disease had a significant transverse sinus stenosis pressure gradient, and 80% proceeded to stenting. The intervention eliminated papilledema in 96% of stented patients, and allowed 81% to cease acetazolamide. The need for a further procedure was low at 6%, and the safety profile was favourable.

**Conclusions:**

Medically refractory disease in IIH is common (18%), and nearly always associated with a significant transverse sinus stenosis pressure gradient (90%). Endovascular stenting of the stenosis deserves wider uptake as a highly effective, safe, and usually definitive treatment. It safeguards vision by eliminating papilledema (96%), and allows most patients to cease acetazolamide (81%). By analogy with glaucoma, if acetazolamide is the prostaglandin of IIH and CSF diversion the emergency glaucoma filter, stenting is the minimally invasive glaucoma surgery.

## Introduction

This paper defines the ophthalmic indications for procedural intervention in an important subset of idiopathic intracranial hypertension (IIH) sufferers. These are adults with medically refractory disease, associated with significant transverse sinus stenosis (stenosis). Such patients have historically progressed to cerebrospinal fluid (CSF) diversion with a shunt (shunting) or optic nerve sheath fenestration (fenestration). Shunting reverses papilledema (1) but is associated with significant morbidity and cost (2), due to infections and the need for repeat procedures (3). Fenestration, while also reversing papilledema (4), is not readily accessed in some regions, and has its own complications (5).

**Figure 1:**
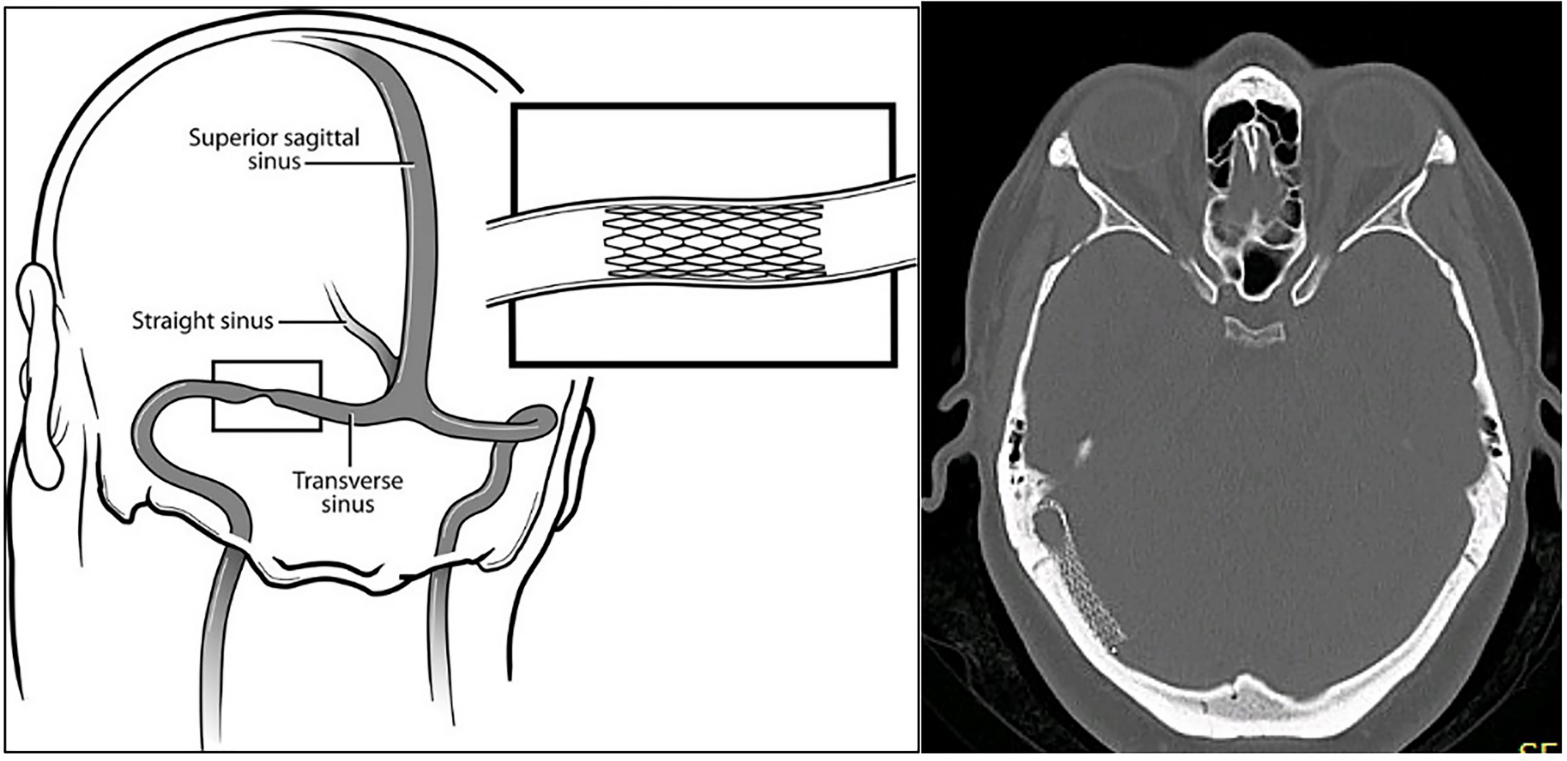
An endovascular stent can be placed to relieve a significant transverse sinus stenosis pressure gradient. (Paul H. Dressel, BFA. Copyright E.I. Levy, published with permission.) The axial CT brain (bone window) with right transverse sinus stent *in situ* clearly shows the stent fenestrations.

The authors show here that transverse sinus stenosis stenting (stenting) in medically refractory IIH offers effective and safe intervention, when visual threat and/or intolerance of maximal medical therapy co-exist with a significant stenosis pressure gradient as measured at catheter venography. Stenting (**Figure 1**) reverses papilledema in such patients by alleviating cerebral venous hypertension. The procedure relieves partial venous obstruction, improving CSF drainage, and hence lowering intracranial pressure.

We present comprehensive ophthalmic data to complement the metrics of catheter venography. Systematic ophthalmic assessment and documentation is key in managing IIH - both in identifying those patients who will benefit from a stent, and in assessing the efficacy of the intervention. Our paper advances the IIH literature through its focus on the ophthalmic aspects of stenting (6).

## Methods

### Ophthalmic Data

The database of all 226 successive IIH patients seen from 2012 to 2021 at Canberra Hospital was reviewed by its sole author, the senior ophthalmologist listed here as first author. Included patients met the criteria for a diagnosis of IIH (7). Adult patients who had received a stent at Royal Prince Alfred Hospital were then identified. The ophthalmic data of the stented patients was tabulated before and after the procedure, noting best corrected visual acuity (BCVA), visual field mean deviation (MD), average retinal nerve fibre layer thickness (RNFL) on ocular coherence tomography (OCT) (8), IIH medication, and time from diagnosis to stenting (**Supplementary Table 1**).

Central acuity was measured on a LogMAR vision chart, but recorded as the Snellen equivalent. Automated perimetry was undertaken on a Humphrey Field Analyser, using a 30-2 protocol. Perimetry was supervised by an experienced orthoptist, and repeated if the reliability indices were unsatisfactory. The MD was taken as a proxy for the extent of field loss. The worst visual field (field) in either eye prior to stenting was selected as a marker of disease severity/visual threat.

RNFL was used to assess the degree of disc swelling. Papilledema was defined as an RNFL ≥ 130 µ (normal 100 µ) in at least 1 eye. At this level, papilledema becomes clinically appreciable, and allowance is made for an RNFL somewhat higher than 100 µ in younger patients. The worst RNFL prior to stenting was selected as a marker of disease severity/visual threat. The RNFL was sometimes less than the normal of 100 µ, if optic atrophy had already developed.

**Figure 2:**
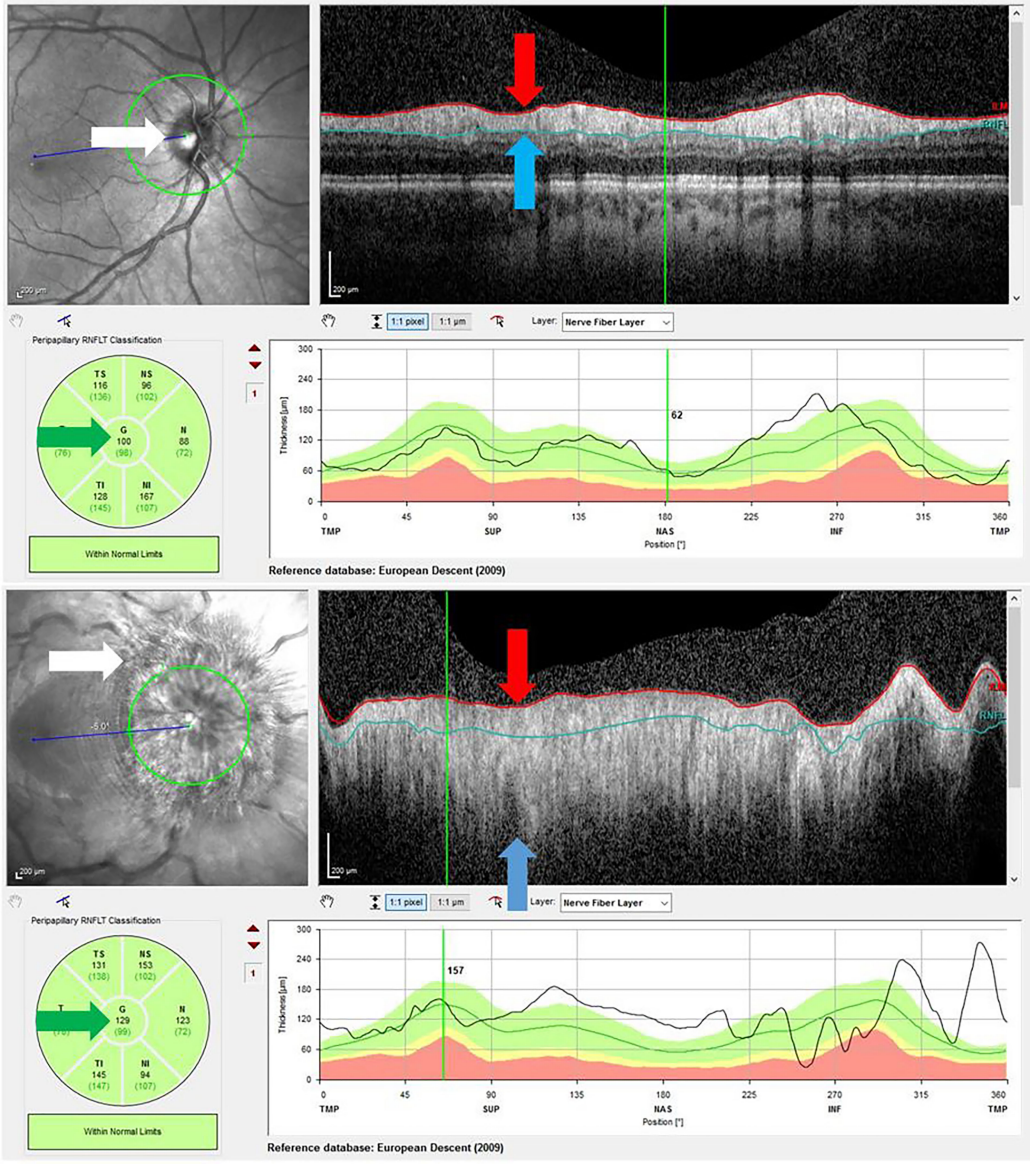
Above – OCT RNFL of normal optic disc. The disc margin is sharp (white arrow), and the RNFL fully contained between the red and blue limit lines (red and blue arrows). The average thickness is 100 μ (green arrow, centre number). Below – OCT RNFL in gross papilledema. The disc margin is obliterated (white arrow), and the RNFL extends far beyond the red and blue limit lines (red and blue arrows). Hence the average thickness measurement of 129 μ (green arrow, centre number) is invalid.

Due to segmentation errors, in patients with severe papilledema the RNFL cannot be measured accurately (9), and the machine-generated RNFL average thickness must be disregarded (**Figure 2**, green arrows). Such RNFLs were recorded as 450 µ rather than ‘unreliable’ (**Supplementary Table 1**). This value was derived by comparing the physical width of the ‘rolled out’ tomogram of the RNFL in a normal patient to that in a patient with gross papilloedema (**Figure 2**, red and blue arrows).

### Medication

Note was made of the IIH medication each patient was taking before and after stenting. The highest dose prescribed at any point during treatment was used as a marker of disease severity /visual threat. Inability to continue with medication due to side effects was noted.

### Determination of Medically Refractory IIH

Medically refractory IIH was diagnosed if there was visual threat, as assessed on field loss and/or papilledema, and/or if there was intolerance of maximal medical therapy (**Table 1**). Patients found to have medically refractory disease went on to catheter venography, to ascertain if there was a transverse sinus stenosis pressure gradient that merited stenting.

**Table 1:** Definition of medically refractory IIH.

Medically refractory IIH
** 1 or more of**
Progressive visual field loss on maximal drug therapy*
Persisting papilledema on maximal drug therapy*
Recurrent papilledema on weaning of drug therapy
Intolerance of maximal drug therapy*
*Acetazolamide 1,000mg daily +/- topiramate up to 200 mg daily.

One patient deemed to have fulminant disease was not considered for stenting. There was such profound papilledema and advanced field loss at presentation that same-day procedural intervention was sought in the form of shunting.

### Lumbar Puncture

All patients receiving a stent had previously undergone lumbar puncture (LP) measurement of CSF opening pressure with CSF analysis, to confirm the diagnosis of IIH. Where multiple measurements were available, the highest CSF pressure was selected as a marker of disease severity.

### Manometry Data From Catheter Venography

**Figure 3:**
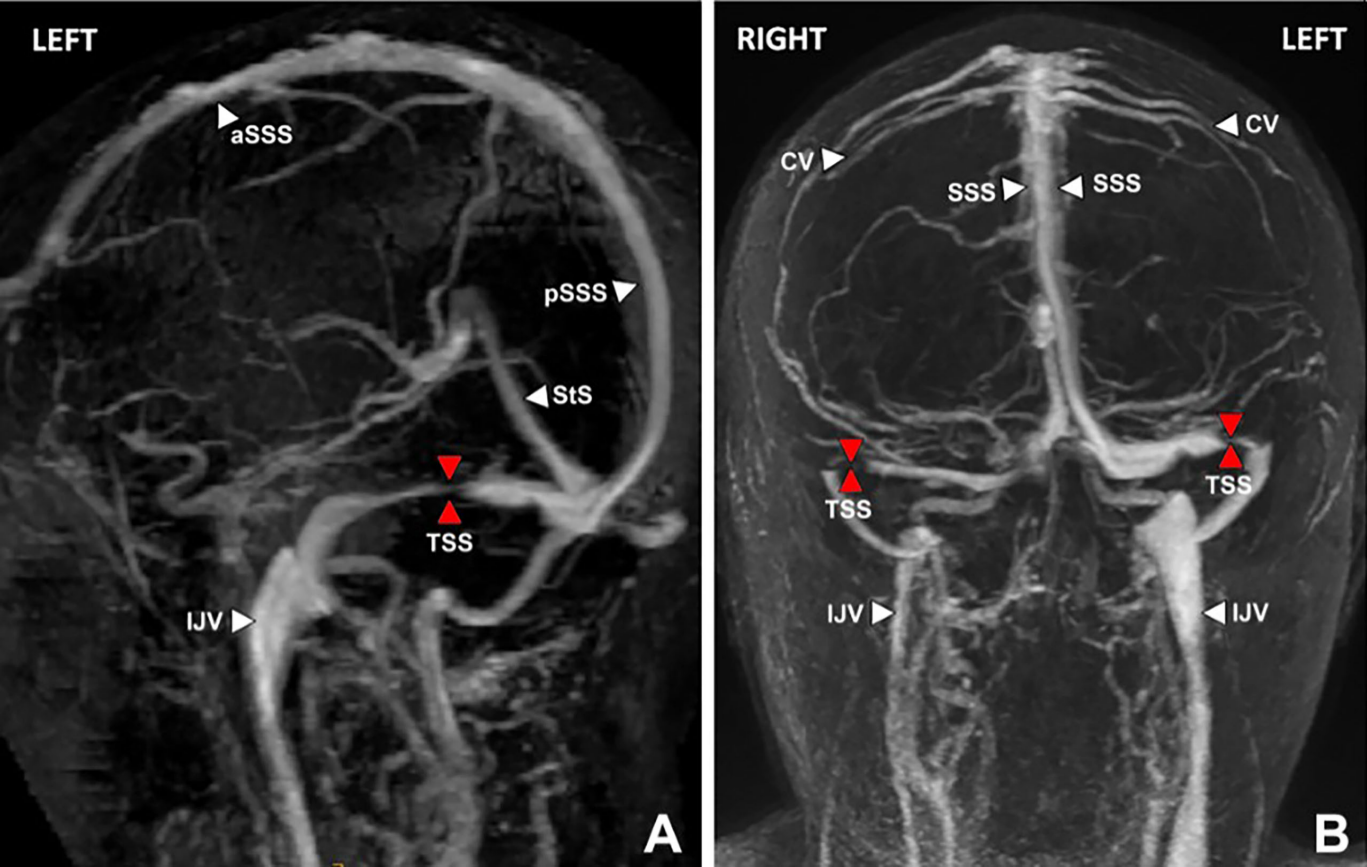
MR venogram brain, lateral oblique **(A)** and posterior views **(B)** showing severe stenosis (red arrows) of the dominant left and non-dominant/hypoplastic right transverse sinuses (TSS). Venous hypertension does not develop unless the dominant transverse sinus is abnormal, or both co-dominant transverse sinuses are affected. Key: aSSS, anterior superior saggital sinus; pSSS, posterior superior saggital sinus; StS, straight sinus; TSS, transverse sinus stenosis; IJV, internal jugular vein; CV, cortical vein.

All patients receiving a stent had initially been found to have bilateral variations in transverse sinus anatomy on CT or MR venography (**Figure 3**). Findings were either bilateral stenosis, or unilateral stenosis with contralateral hypoplasia. Such findings led to assessment with catheter venography of the dominant transverse sinus by a senior neuro-interventionalist. This investigation was performed with the expectation that if a significant stenosis gradient was detected, then it would be treated with stenting in preference to shunting or fenestration.

Two weeks prior to catheter venography, patients had either ceased or halved their acetazolamide, to solve the therapeutic dilemma of recording an equivocal stenosis gradient whilst on high-dose medical treatment – while the plasma half-life of acetazolamide is 4-8 hours, its pharmacologic effect may last longer (10). Topiramate was not adjusted, given that sudden withdrawal is not recommended, albeit in the context of its use in epilepsy (11).

LP was also avoided prior to venography for at least 2 weeks, as the abrupt lowering of CSF pressure which follows LP may persist even some weeks later. (An LP was sometimes performed immediately after venography, providing immediate treatment if there was concern about the visual status.)

The catheter venography studies were reviewed for the absolute pressure in the superior sagittal sinus (SSS) using the right atrial pressure as a reference point. The relative gradient across the stenosis in the dominant transverse sinus was then measured. A significant stenosis gradient was defined as a minimum of 8 mmHg, for a normal drop in pressure across the transverse sinus of ≤ 4 mmHg. (Note that venography measurements are obtained in mmHg, while lumbar puncture results use cmH2O. Multiplying mmHg by 1.36 converts this unit to cmH2O.)

**Figure 4:**
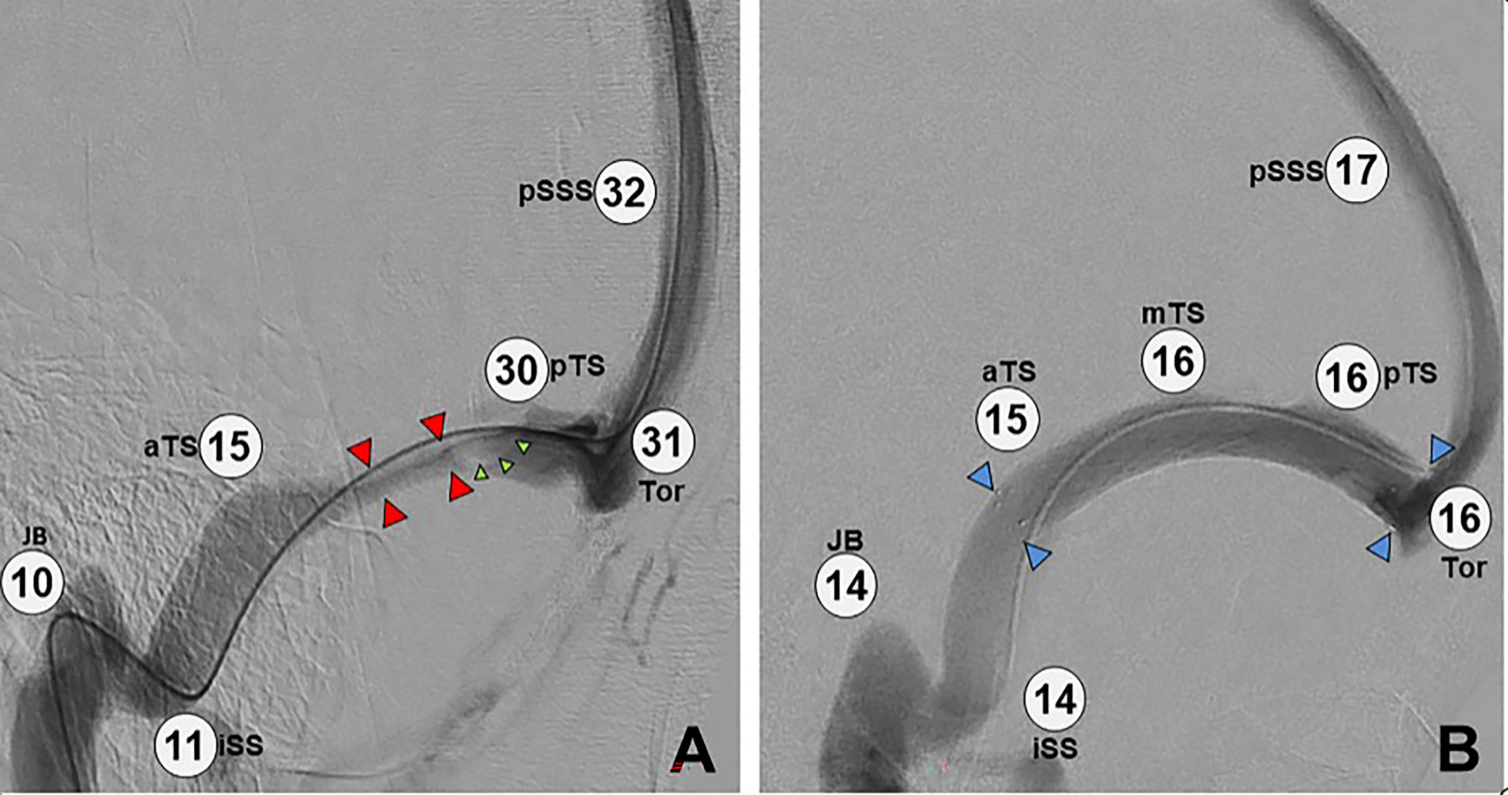
Image intensifier views before and after placement of a transverse sinus stent. **(A)** Catheter cerebral venography and manometry demonstrating elevated overall pressure (pSSS 32 mmHg) and a long segment of extrinsic stenosis in the left transverse sinus (red arrows), plus a large arachnoid granulation (green arrows). Note the large pressure gradient across the stenosis (30 mmHg – 15 mmHg = 15 mmHg). **(B)** Post-stenting study in the same patient, demonstrating overall lower pressure (pSSS 17 mmHg), a patent stent (blue arrows) with resolved stenosis, and no significant pressure gradient (16 mmHg – 15 mmHg = 1 mmHg). Key: pSSS, posterior superior saggital sinus; pTS, posterior transverse sinus; Tor, torcula; aTS, anterior transverse sinus; JB, jugular bulb; iSS, inferior saggital sinus; mTS, middle transverse sinus.

Immediately after stenting of a significant stenosis, dramatic alteration is seen in the venous anatomy (12), with reduction of the pressure gradient to the normal range (**Figure 4**).

### Data Quality

The database of 226 consecutive patients diagnosed with IIH in Canberra Hospital’s Ophthalmology Service was compiled by the sole treating senior ophthalmologist and first author, using a standardised template. The entire database was scrutinised to ensure that no patient had disc drusen alone, an alternative cause of papilledema such as cerebral venous sinus thrombosis, or an equivocal diagnosis of IIH in retrospect.

The data regarding stented patients was comprehensive (**Supplementary Table 1**), with only 29 of 768 ophthalmic and neuro-radiologic data points unavailable or unreliable. All stenting was carried out at one centre by senior neuro-interventionalists. All patients in the IIH database who were stented at Royal Prince Alfred Hospital were included. The sample size of stented patients was reasonable at 32, given that a 2012 meta-analysis of 8 case series and 7 case reports yielded a sample size of 143 (13).

Potential causes of error in data acquisition included patients feeling unwell and so providing an inaccurate field; inaccurate LP measurements due to obesity and positioning issues; and segmentation errors in the RNFL for very swollen discs, as discussed above.

## Results

**Figure 5:**
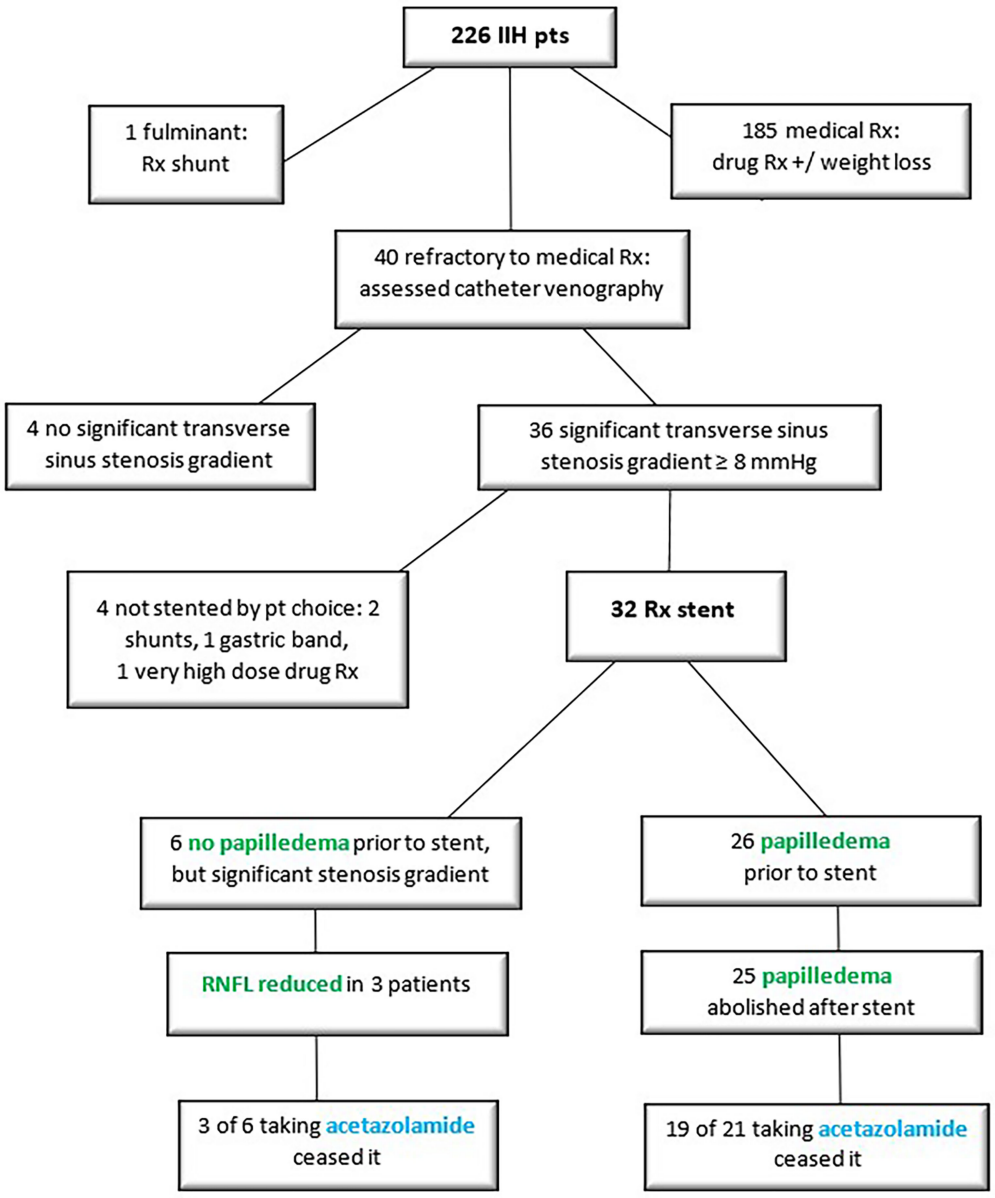
Flow chart of main outcomes in stented patients.

Medically refractory IIH was found in 18% of the total cohort of IIH patients. Of those, 90% with medically refractory disease had a significant transverse sinus stenosis pressure gradient, and 80% proceeded to stenting. The intervention eliminated papilledema in 96% of stented patients, and allowed 81% to cease acetazolamide. The need for a further procedure was low at 6%, and the safety profile was favourable (**Table 2**, **Figure 5**).

**Table 2:** Main outcomes in stented patients.

Outcome	Number	Percentage
Number of patients with medically refractory IIH	40/226	18%
Medically refractory IIH with significant stenosis	36/40	90%
Medically refractory IIH receiving a stent	32/40	80%
Resolution of papilledema after stenting	25/26	96%
Cessation of acetazolamide after stenting	22/27	81%
Post-stent procedure	2/32	6%
Severe complications	0/32	0%

Additional Results are recorded in **Table 3**.

**Table 3:** Additional outcomes in stented patients.

Parameter	Normal	Mean	Range	Comments					
Sex [Male/female]	5/6 or 83% of all male pts in the total IIH database received a stent 27/220 or 12% of all female pts in the total IIH database received a stent								
**Stent data**									
Age at stenting [Years]	N/A	31.2	17 - 46						
LP opening pressure [cm H2O]	20 - 22	37.1	24 – 84						
SSS pressure [mmHg]	10 - 15	33.5	21 - 65	Multiplying mmHg by 1.36 converts this unit to cmH2O.					
Transverse sinus stenosis gradient [mmHg]	≤ 4 (no stenosis)	17.2	8 - 40						
**OCT RNFL**									
Worst before stent [µ]	100	248	87 - 450	Papilledema defined as > 130 µ; RNFL defined as 450 µ in gross disc swelling /unreliable OCT.					
After stent [µ]	100	101	71 -164						
**Visual acuity **									
Pre-stent [m]	6/6	6/6	6/4 – 6/12						
Post-stent [m]	6/6	6/6	6/4 – 6/15						
**Visual field mean deviation**									
Pre-stent [dB]	0 to -1.0	- 4.43	- 15.20 to - 0.27						
Post-stent [dB]	0 to -1.0	- 1.45	- 4.77 to + 1.36						
**Medical treatment**									
Duration medical Rx prior to stent [months]	N/A	24	1 - 84	Periods of management by other doctors excluded. Median 22 months.					
Weight loss prior to stent [kg]	N/A	10.5	1-27	Weight loss prior to stent in 14/32 pts.					
**Acetazolamide**									
Pre-stent [mg]	N/A	1,176	750 - 1,750	5 pts ceased acetazolamide prior to stenting due to side-effects.					
Post-stent [mg]	N/A	48	0 - 500						
**Topiramate**									
Pre-stent [mg]	N/A	69	0 - 200	5 pts ceased topiramate prior to stenting due to side-effects, and 9 were not prescribed it. These pts are excluded. 2 pts continued topiramate for migraine.					
Post-stent [mg]	N/A	10	0 - 200						
**Follow-up after stent** [months]	N/A	19	0 - 56	Follow-up < 2months for 5 pts due to recent procedure.					

NA, Not applicable.

### Complications of Stenting

A low complication rate was observed in the stented group (**Table 4**). The only significant complication was the development of mural change within the stent in 2 patients. A differential diagnosis of thrombosis was considered, but anticoagulation was ceased when the appearance was static on serial imaging. A final diagnosis of ingress of an arachnoid granulation through the mesh of the stent was made in both cases.

**Table 4:** Complications of stenting.

COMPLICATIONS OF STENTING	
POTENTIAL	OBSERVED
**1. From catheter venography**	
Injury to venous system	Nil
Neurological deficit	Nil
Vasovagal	1 pt
**2. During stent placement**	
Death	Nil
Neurological deficit	Nil
Injury to venous system	Nil
Vasovagal	1 pt, transient self-limiting asystole
Complications of general anaesthesia	Nil
**3. After stent placement**	Nil
Intraluminal stent thrombosis	Nil
Mural change in stent	2 pts, see text for more detail
Significant bleeding on dual antiplatelets	Nil (outstanding surgery scheduled before stent)
Transient headache post-stent	Nearly universal, self-limiting

While headache immediately after stenting due to dural stretch was nearly universal, it was self-limiting within a few weeks.

The complication rate here compared favourably with a 2021 multicentre database study (14), which identified 6 major complications from a total of 811 stents and 1,466 catheter venograms for IIH. These authors found 1 fatality after stenting, equating to a mortality of 0.1%. To place this in context, mortality of CSF shunting in children for hydrocephalus was around 0.5% in a study of 5,955 patients in the US between 1998 and 2000 (15). The high failure and complication rates associated with CSF shunting are referenced above.

### Subsequent Procedures

Of the 32 stented patients, 2 went on to a second procedure. Although 2 demonstrated restenosis adjacent to the stent, only 1 needed a second stent. Another patient proceeded to a shunt for persistent elevation of SSS pressure, despite abolition of her transverse sinus stenosis pressure gradient by stenting.

## Discussion

Escalating papilledema is eventually blinding (16) and must therefore be reversed. This should ideally be done while it is still pre-perimetric but certainly before there has been severe field loss. Cerebral venous hypertension is now understood to be a key element in papilledema (17), as it reduces the pressure gradient between the CSF compartment and the venous sinuses into which CSF ultimately drains. Hence, venous hypertension leads to raised CSF pressure. A negative feedback loop is established, in which rising CSF pressure compresses the cerebral venous sinuses further, perpetuating the venous hypertension.

In more severe IIH, this cerebral venous hypertension is nearly always accompanied by transverse sinus stenosis. Whether the stenosis is intrinsic or secondary to raised intracranial pressure, stenting of a significant stenosis obliterates the stenosis, relieves the venous hypertension, improves CSF drainage, and so reverses papilledema (18). Stenting is now increasingly recognised in managing medically refractory IIH (19–21).

### Patient Selection for Stenting

#### Visual Threat

Selection for stenting first requires identification of medically refractory disease, one element of which is visual threat. Note that central visual acuity is preserved in papilledema until damage to the optic nerve is very advanced, due to relative sparing of the maculopapillar bundle (22). The visual field is thus the most important quantifier of visual threat (23, 24).

#### Intolerance of Maximal Medical Therapy

In this study, a patient was considered refractory to medication on failing treatment with acetazolamide ≥ 1,000 mg daily, with the average dose a burdensome 1,176 mg daily (750–1,750mg). Use of acetazolamide in higher doses proved unacceptable to most of our patients due to its side-effect profile (25, 26), although the literature does report tolerability of such doses (27). Bloody diarrhoea, and inability to maintain previous employment or studies due to cognitive change, were the most problematic issues.

The maximum dose of topiramate used was 200 mg, although not all patients were prescribed it. The effects of topiramate on contraception (28) and pregnancy (29) were amongst the concerns limiting the use of this medication.

Despite the issues with medication side-effects, a quite lengthy mean of 24 months (1-84 months, median 22 months) of drug therapy was undertaken prior to stenting. This allowed the patient sufficient time to make every effort to lose weight. It also gave time for a thorough assessment of various dosing regimens. Thus, failure of conservative treatment was truly proved before considering invasive work-up and procedural intervention.

As in glaucoma, high-dose acetazolamide is not a sustainable long-term treatment plan. The patient who complies with high-dose medication experiences a significant decline in quality of life, while the patient who does not comply remains at risk of permanent visual loss.

#### Stenosis Gradient

The definition of a significant stenosis gradient in this study of ≥ 8 mmHg equalled (30) or approximated (31) the criteria used in similar case series. The average stenosis gradient was found to be 17.2 mmHg (8–40 mmHg), a little lower than the 21 mmHg identified in a comparable study (32).

**Figure 6:**
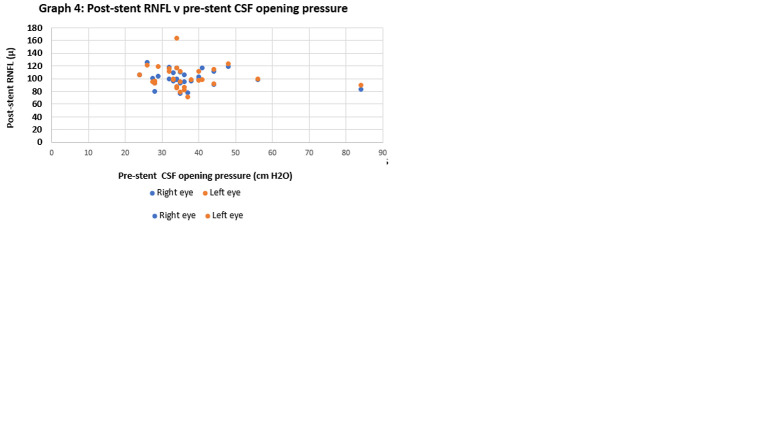
Negative correlations amongst LP opening pressure, RNFL and stenosis gradient.

Stenosis gradient did *not* show any correlation with CSF opening pressure or pre-stent RNFL (**Figure 6**). Thus, catheter venography should still be considered in a patient whose clinical course suggests medically refractory IIH, even if the LP pressure and/or RNFL are unimpressive.

IIH without papilledema is well recognised (33). In this cohort, 6 patients did not have papilledema as we numerically defined it. They did have abnormal visual fields, medication intolerance or both, as well as a compelling stenosis gradient leading to stenting. In 3 patients, the RNFL declined after stenting. Bearing in mind that the RNFL is the sum of swelling plus atrophy, we surmise that they already had some optic atrophy at the time of stenting.

#### Assessment Over Time

Sequential assessment of the clinical gestalt over time improves the identification of patients with medically refractory disease. It is important to develop a consistent data set that is updated at each consultation, collating RNFL, fields, fundoscopy, medication, weight, and patient symptoms. This multimodal tracking ensures timely detection of clinical trends that require a change in management.

In quantify to assess the amount of papilledema, objective quantification of RNFL thickness with OCT is much more reliable than subjective Frisen grading of the disc appearance (34). Nevertheless, stereoscopic examination of the disc does provide qualitative information regarding the presence or absence of pallor. Pallor is an indicator of emerging optic atrophy, even if the disc is still swollen.

As in glaucoma, the longitudinal tracking of optic nerve *structure* (RNFL) and *function* (visual fields) guides the recommendation to intervene, rather than a rigid numeric rubric. It is perfectly reasonable to trial medical therapy for a grossly swollen pink disc if the visual field shows only mild to moderate blind spot enlargement, and compliance with medication and follow-up is assured. However, if the visual fields show gross blind spot enlargement with significant peripheral loss, or if optic disc pallor is already evident, then early consideration should be given to catheter venography to prevent/minimise permanent structural damage to the optic nerves.

### Efficacy of Stenting in Eliminating Papilledema

In this cohort of patients, average RNFL declined by 147 µ (from 248 to 101 µ) after stenting, with resolution of papilledema in 96%. This accorded with a 2018 meta-analysis of 474 stented patients, which demonstrated improvement of papilledema in 94% (35).

Since RNFL is a proxy for intracranial pressure (36), the impressive decline in RNFL after stenting seen here indicated not only resolution of papilledema, but also resolution of IIH. However, a critical caveat in assessing reduction of papilledema is that a declining RNFL is good news only if the visual field remains stable or improves. If the field is declining along with the RNFL, the patient actually has uncontrolled disease. Since the RNFL is the sum of swelling plus atrophy, the stereoscopic examination of the optic disc for swelling is therefore an important part of interpreting the RNFL and field.

### Weight Loss Prior to Stenting

Weight loss is considered a major part of treatment in IIH, as it reduces central venous pressure, and may reverse obesity-related inflammation (37) and metabolic changes (38) implicated in the aetiology of the condition. However, many patients find it impossible to lose and then keep off the recommended minimum 10% of body weight (39). Here only 44% of patients achieved weight loss prior to stenting, with an average loss of 10.5 kg. This amount was not sufficient to achieve clinical remission, even though 72% of patient in another study did achieve remission for this quantum of weight reduction (40).

Those patients not identified in this cohort as losing weight prior to stenting were a heterogenous group, including those of normal weight, those losing no weight or losing it after stenting, those gaining weight, and those keeping incomplete records.

Many things contribute to difficulties with achieving weight loss. Obesity often carries a history of dieting followed by rebound in weight (41), as well as complex psychodynamic issues around body image (42). Bariatric surgery, while superior to community weight loss programs at 2 years (43), is far from readily obtained in the public health system, and too expensive in the private health system for most patients. Even when weight loss is achieved, it does not always produce the desired remission in IIH, as evidenced by the patients in this study who did lose weight prior to procedural intervention. Stenting offers a way out of these multiple impasses.

### Visual Outcomes

While the average visual acuity was unaltered by stenting, the average visual field mean deviation improved by 2.88 dB: from -4.43 dB before (-15.20 to -0.27 dB) to -1.45 dB after (-4.77 to +1.36). This was double the 1.43 dB improvement due to acetazolamide alone seen in the 2014 IIH Treatment Trial for patients with mild IIH (44). In our series, 47 of 48 eyes showed improvement in the field mean deviation.

### Patient Gender

In this IIH database of 226 patients, 5 of the 6 adult men proved to have medically refractory disease requiring stenting. This is in line with other studies confirming that IIH is more aggressive in men (45), with severe visual loss twice as likely compared to women.

None of the women who went on to stenting presented during pregnancy. Hence there was no need to avoid iodinated contrast and radiation in diagnosis and treatment, as would usually be the case in pregnancy. With a stent in situ, 1 patient went on to 2 uncomplicated pregnancies.

### Avoiding Complications During Stenting

Safe stenting starts with co-ordination of care amongst the patient’s ophthalmologist, neurologist, and neuro-radiologic interventionalist, to ensure correct patient selection. Indications for stenting are summarised in **Table 5**. Appropriate device selection is a fundamental aspect of a safe procedure, but is beyond the scope of this paper. Other important considerations are appropriate training and experience for the interventionalist and for the anaesthetist.

**Table 5:** Indications for stenting.

Indications for stenting
Adult patient with
** Medically refractory IIH: 1 or more of**
Progressive visual field loss on maximal drug therapy*
Persisting papilledema on maximal drug therapy*
Recurrent papilledema on weaning of drug therapy
Intolerance of maximal drug therapy*
** AND**
Significant transverse sinus stenosis ≥ 8 mmHg
** AND**
Ability to comply with antiplatelet therapy for 1 week before and 1 year after stenting
* Acetazolamide 1,000mg daily +/- topiramate up to 200 mg daily

### Prevention of Stent Thrombosis

Acute intraluminal stent occlusion due to thrombosis would be potentially life-threatening in a patient who, by definition, has an inadequate contralateral transverse sinus. However, stent thrombosis was not seen in this series, and appears to be extremely rare in patients managed with dual antiplatelet premedication (46). Here all patients were prescribed dual antiplatelets for 1 week prior to stenting, nearly always aspirin 100mg and clopidogrel 75 mg daily. Suppression of platelet function was ascertained immediately prior to the procedure, and a heparin bolus was used on the table. Both anti-platelets were continued for 3 months post-stent, and aspirin was used for another 9 months thereafter. This long duration of antiplatelet medication allows time for endothelium to fully line the stent.

Patients who might struggle to comply with the antiplatelet regime, who will need surgery in the year following stenting, or who are at risk of being lost to follow up in the first year after treatment, should not be offered a stent.

### Informed Consent

To facilitate informed consent, catheter venography was carried out as a stand-alone procedure some weeks prior to stenting. It is the authors’ experience that patients are eager for procedural intervention when their vision is threatened, or after many months of unpalatable medication. This makes the need for thorough education about the potential risks of the procedure especially important. Patients considering stenting are counselled that it carries around a 1% risk of serious complication, including stroke and death. More recent literature suggests that the risk is significantly lower than this, as discussed above.

### Stenting in Fulminant IIH

Stenting is gaining ground in patients with fulminant disease, defined in one paper as visual field loss to within 5 degrees of fixation, and/or a decrease in visual acuity to ≤ 6/15 in either eye in the presence of papilledema (47). However, the sole fulminant presentation to the first author was not considered for stenting, due to the delay imposed by obtaining catheter venography at another centre and the requirement for a week of antiplatelet therapy prior to stenting. Additionally, it was felt that the commencement of antiplatelet therapy would mean that if a stent proved an inadequate intervention, it would not be possible to perform a lumbar puncture/drain or shunt on an emergency basis.

### Stenting Compared to Fenestration and CSF Shunting

There are currently no head-to-head prospective or randomised control trials assessing stenting relative to other procedural interventions. However, meta-analyses comparing them (48–50) do position stenting favourably, due to its high technical and clinical success rates, and low rate of complication.

Since few treatment centres offer all three options of shunting, fenestration, and stenting, it may be difficult to construct trials comparing the three. Nevertheless, as the prevalence of IIH increases (51) along with obesity (52), research opportunities may increase too.

The authors propose that because stenting is a highly effective and safe treatment, it is preferable to shunting. Shunting carries the risks of haemorrhage, infection, epilepsy, and focal neurological deficit. Nor is placement of a shunt technically straightforward in IIH patients, with slit-like ventricles as part of their disease (53). Complications from placing shunt tubing in the abdomen may also be increased by obesity.

The most significant issue with shunts, however, is that they frequently require surgical revision. The average time to revision is a brief 6 months for some 19% of patients (54). Revision rates overall are quoted at 25% within the first year, and up to 85% within the patient’s lifetime (55). Shunt failure may be due to infection, mechanical malfunction, or unsatisfactory rates of CSF drainage, whether excessive or inadequate.

Fenestration, while a proven therapy to protect vision in fulminant and medically refractory IIH (56), is not readily available in some regions. Stent placement by comparison may be more easily accessed. The advent of clot retrieval for large vessel stroke has increased the number of neuro-interventionalists in tertiary hospital imaging departments who can acquire the appropriate training and experience to undertake stenting. The tertiary setting provides neurosurgical back-up, in the very unlikely event of an intracranial complication. Anaesthetic and high dependency services are also available, for the requisite general anaesthesia and post-procedure observation. In summary, stenting avoids the risks associated with shunting, and the limited availability of fenestration.

### Limitations of the Study

Headache was deemed too subjective and multifactorial (57) a symptom to be assessed in this paper.

However, headache certainly creates very significant disability for IIH sufferers (58). When responding to a questionnaire about their symptoms, only 18.1% of IIH patients indicated that their lives were most affected by visual problems, with 49.6% most affected by headaches (59).

It has been mentioned above that a declining RNFL must always be interpreted in the context of the visual field to ascertain that the decline represents clinical recovery and not optic atrophy. However, given that significant RNFL thinning can be pre-perimetric (as seen in glaucoma), it is possible that an RNFL decline may have elements of both resolution of swelling and onset of pre-perimetric atrophy. Since stereoscopic assessment of the disc for pallor and swelling is subjective, it would be ideal to have an objective measure of pre-perimetric optic atrophy. Nevertheless, the authors conclude that RNFL decrease in the presence of a normal or stable field is clinically acceptable evidence that vision is protected.

## Conclusion

Adults with IIH who fail medical therapy nearly always have a significant transverse sinus stenosis pressure gradient. In such patients, stenting safely and very effectively reverses papilledema. In addition, the majority of patients are able to discontinue medication. Timely stenting protects vision, and improves quality of life. By analogy with glaucoma, if acetazolamide is the prostaglandin of IIH and CSF diversion the emergency glaucoma filter, stenting is the minimally invasive glaucoma surgery (MIGS).

## Data Availability Statement

The original contributions presented in the study are included in the article/**Supplementary Material**. Further inquiries can be directed to the corresponding author.

## Ethics Statement

This study involving human participants was reviewed and approved by the ACT Health Human Research Ethics Committee, Low Risk Sub-Committee. Written informed consent for participation was not required for this study, in accordance with the national legislation and the institutional requirements.

## Author Contributions

KR compiled all data and drafted the manuscript and figures. GP, TA, and SW carried out the venography, manometry and stenting, and provided the venous pressure data. SW and MH prepared the radiologic images. GP and MH provided critical revision of the manuscript. All authors approved the submitted version.

## Conflict of Interest

The authors declare that the research was conducted in the absence of any commercial or financial relationships that could be construed as a potential conflict of interest.

## Publisher’s Note

All claims expressed in this article are solely those of the authors and do not necessarily represent those of their affiliated organizations, or those of the publisher, the editors and the reviewers. Any product that may be evaluated in this article, or claim that may be made by its manufacturer, is not guaranteed or endorsed by the publisher.
